# A Mass‐Spectrometry‐Based Modelling Workflow for Accurate Prediction of IgG Antibody Conformations in the Gas Phase

**DOI:** 10.1002/anie.201812018

**Published:** 2018-11-27

**Authors:** Kjetil Hansen, Andy M. Lau, Kevin Giles, James M. McDonnell, Weston B. Struwe, Brian J. Sutton, Argyris Politis

**Affiliations:** ^1^ Department of Chemistry King's College London 7 Trinity Street London SE1 1DB UK; ^2^ Waters Corp. Stamford Road Wilmslow SK9 4AX UK; ^3^ Randall Centre for Cell and Molecular Biophysics King's College London UK; ^4^ Department of Chemistry University of Oxford UK

**Keywords:** conformation analysis, immunoglobulin, ion mobility, mass spectrometry, molecular dynamics

## Abstract

Immunoglobulins are biomolecules involved in defence against foreign substances. Flexibility is key to their functional properties in relation to antigen binding and receptor interactions. We have developed an integrative strategy combining ion mobility mass spectrometry (IM‐MS) with molecular modelling to study the conformational dynamics of human IgG antibodies. Predictive models of all four human IgG subclasses were assembled and their dynamics sampled in the transition from extended to collapsed state during IM‐MS. Our data imply that this collapse of IgG antibodies is related to their intrinsic structural features, including Fab arm flexibility, collapse towards the Fc region, and the length of their hinge regions. The workflow presented here provides an accurate structural representation in good agreement with the observed collision cross section for these flexible IgG molecules. These results have implications for studying other nonglobular flexible proteins.

Immunoglobulins (Ig), or antibodies, are the proteins responsible for mediating an extensive network of immunological responses. The past decades have seen a steady increase of interest in developing Igs as biotherapeutic agents for the treatment of various diseases, including cancer and autoimmune disorders.^1^, ^2^, ^3^ While the architectures of Igs are relatively conserved, they exhibit dramatic differences in their dynamics and mode of interactions with antigens and cognate receptors.^4^, ^5^ These differences stem from intrinsic features in their structures such as binding‐site specificity and hinge flexibility (Figure 1 a).^6^

**Figure 1:**
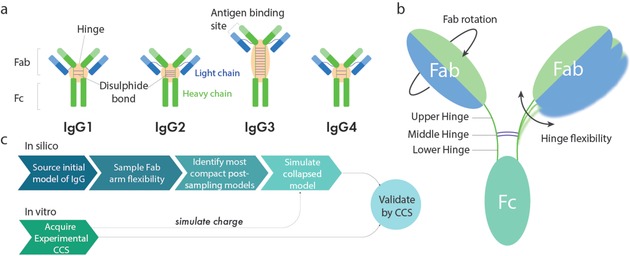
Schematics and workflow for modelling antibody flexibility. a) Schematic representation of human IgG1–4 subclasses. b) Representative structure of IgG1, denoting hinge substructure and modes of Fab movement stemming from the upper hinge. c) Integrative workflow generating and comparing the calculated CCS values of initial, post‐sampling, and gas‐phase MD models with experimental CCS values.

There are five isotypes or classes of Igs, the most abundant of which in humans is Ig gamma (IgG), comprising approximately 75 % of all human antibodies in serum.^7^ IgG is by far the most commonly exploited isotype for biotherapeutics,^1^ including bispecific antibodies^8^, ^9^ and antibody–drug conjugates (ADCs).^10^, ^11^, ^12^ In 2017, ten new antibody therapeutics were approved, all of which were IgG‐based.^13^ There are four subclasses of human IgG, named IgG1–4 (Figure 1 a). While IgG1, 2, and 4 are similar in topology, overall length, and hinge length, IgG3 has a markedly longer hinge, producing a molecule much longer than the other subclasses.^14^, ^15^ IgG molecules exhibit a high degree of heterogeneity because of their extensive glycosylation, and also sequence variability in their antigen binding regions. All IgG molecules consist of two heavy chains and two light chains that are covalently linked via disulphide bridges in a characteristic “Y” shaped topology (Figure 1 b). A central hinge separates two Fab “arms” from the Fc “stem” of the IgG molecule. This hinge plays a pivotal role in providing IgG molecules with flexibility, allowing relative Fab–Fab and Fab–Fc movements.^16^ The hinge and Fc region play an important role in binding immune effector proteins including, the Fc gamma receptors (FcγR), neonatal Fc receptor (FcRn), and complement component C1q^14^ (Figure 1). The ability for all IgG subclasses except IgG4 to trigger the complement cascade via C1q,^17^ for example, illustrates that the intrinsic structure and dynamics of these molecules have functional consequences for each of the IgG subclasses.

Native mass spectrometry (MS) has recently emerged as a powerful method for interrogating proteins and their complexes, providing valuable information about their stoichiometry and topology.^18^, ^19^, ^20^, ^21^, ^22^, ^23^, ^24^, ^25^ Native MS can be hyphenated with IM; the resulting ion mobility (IM)‐MS method offers an extra dimension enabling shape information on the investigated proteins. IM‐MS allows for derivation of topological information of proteins through calculating their collisional cross section (CCS). CCS is described as the rotationally averaged cross section of a molecule and is calculated based on the overall size and molecular architecture.^26^ The experimentally measured CCS can be compared to theoretical CCS calculated from structural models derived by molecular dynamics (MD) simulations or other modelling techniques,^27^, ^28^ thus enabling structures to be assigned back to experimental observations.^29^

Native MS mainly uses electrospray ionisation (ESI) for the purpose of creating multiply charged protein ions.^30^ The response of folded proteins entering the gas phase through ESI is most commonly described through the charged residue model (CRM).^31^, ^32^, ^33^ The CRM envisions gradual droplet desolvation leading to production of a dry protein ion. While the behaviour of a globular protein transferring into the gas phase of a mass spectrometer can be rationalised under the CRM framework, here we pose the following question: do these same rules apply to nonglobular and flexible proteins? Early studies which compared the experimental CCS of antibodies to those calculated from their crystal structures, observed a greater than 30 % discrepancy between these CCS values,^34^, ^35^ thus suggesting collapse of the protein in the gas phase. Such collapse is experienced by nonglobular molecules that are intrinsically flexible or disordered in solution and are capable of conformational change.^36^, ^37^, ^38^, ^39^ Whilst others have explored simulating such collapsing structures,^37^ these call for computationally complicated methods such as “trajectory stitching”^40^ or including mobile proton algorithms,^41^, ^42^ which may be impractical for large molecules. Here, we have developed an integrative IM‐MS‐based strategy that enables the prediction of the structure and dynamics of IgG molecules in the gas phase, including, for the first time, capturing and simulating the dynamics of human IgG3 (Figure 1 a–c). In the first step, homology models of the antibodies were built and subsequently subjected to Fab arm sampling, allowing representation of their intrinsic flexibility as a conformational ensemble. Simultaneously, we subjected IgG1–4 to IM‐MS experiments and derived their corresponding CCS values. The most compact conformations were taken forward for vacuum MD simulations in order to model the gas‐phase structure of IgG molecules.

The flexible nature of IgG molecules means that there are currently only four intact IgG crystal structures available: IgG1 (human: 1HZH, mouse: 1IGY),^43^, ^44^ IgG2 (mouse: 1IGT)^45^ and most recently, IgG4 (human: 5DK3).^46^ We modelled human IgG1, 2, and 4 using the available crystal structures, followed by generation of any missing residues (see Methods in the Supporting Information). IgG3, however, presented a greater challenge resulting from its complex hinge structure and lack of crystallographic representation. The absence of an IgG3 intact crystal structure is likely due to its greater flexibility. We thus built a homology model of human IgG3 and subjected it to 100 ns of explicit solvent simulation to model its average solution state conformation (Supplementary Figure 3, Methods). This average conformation exhibits a hinge length of approximately 70 Å between the Fab and Fc regions, with the fully extended length of the hinge at approximately 110 Å (Supplementary Figure 4). This is in agreement with an earlier electron microscopy study by Roux et al. who observed a distance of (80±23) Å between the Fabs and Fc in solution, and an estimated 100–110 Å for the length of the extended hinge,^6^ as well as with other hydrodynamic and solution X‐ray scattering studies.^47^, ^48^

We next subjected all four of the human IgG subclasses to IM‐MS, which allowed us to quantify their topology through their experimental CCS values (Figure 2 a, Supplementary Figures 5, 6, and Table 1). Consistent with previous studies of human IgG1–4,^34^, ^35^ we observed an approximately 30 % difference between the experimental CCS (CCS_exp_) and model CCS (CCS_model_; Table 1), thus suggesting significant structural collapse. Despite the much longer length of IgG3 compared to IgG1, 2, and 4, all IgG antibodies are equally able to collapse, producing a CCS of approximately 7000 Å^2^. We further deglycosylated all antibodies and subjected them to IM‐MS (Supplementary Figures 7, 8)—the resulting CCS showed no significant difference compared to their glycosylated counterparts (<1 %; Supplementary Figure 9 and Methods). To further exclude the possibility that glycoform heterogeneity influences IgG gas‐phase conformations, we characterised the glycans bound to two additional monoclonal antibodies, Herceptin and Waters mAb, and compared their glycoforms to Sigma IgG1 (Supplementary Figure 10 and Methods). While we identified different N‐linked glycans bound to the IgG molecules, their corresponding CCS values were found to be within 1 %, thus indicating no significant changes in their conformations.

**Figure 2:**
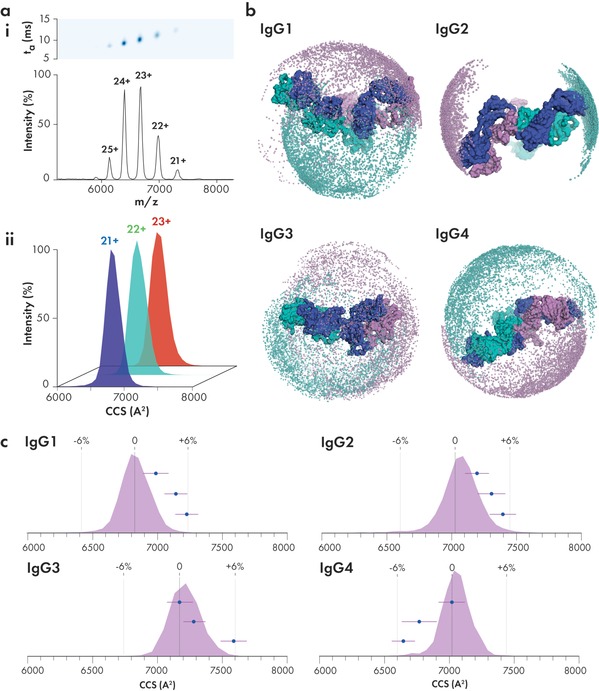
Modelling the conformational flexibility of antibodies. a) Representative mobilogram and native mass spectrum (i) and CCS distributions for 21–23+ charge states of IgG2 (ii). b) Space occupied by IgG1–4 Fabs following upper‐hinge flexibility sampling. Each sphere represents one model for each IgG Fab heavy chain (teal and purple). Light chains are shown as blue. Initial models are shown as surface representations. c) Overlay of experimental CCS distribution with triplicate simulated collapse models. Experimental error is represented by the ±6 % dotted lines. Purple error bars represent the CCS range over the last 1 ns of gas‐phase simulation.

**Table 1:** Experimental and modelling values for IgG1–4.

Subclass^[a]^	IgG1	IgG2	IgG3	IgG4
Theoretical mass [kDa]^[a]^	150	150	170	150
Experimental mass [Da]^[b]^	149 328(±89)	154 297(±42)	162 123(±4)	155 758(±62)
Experimental charge^[c]^	21+	21+	22+	21+
Overall hinge length^[d]^	12	12	62	12
No. hinge disulphides	2	4	11	2
Upper hinge residues sampled	5	3	12	7
Initial model CCS [Å^2^]^[e]^	9532	9747	10 958	9512
Fab arm sampling CCS [Å^2^]^[f]^	8756	8597	9170	8484
ΔCCS of sampling [Å^2^]^[g]^	1102	929	1329	1080
Collapsed model CCS [Å^2^]^[h]^				
Model 1	7226(±176)	7396(±201)	7284(±173)	7017(±204)
Model 2	6988(±196)	7197(±184)	7176(±197)	6766(±268)
Model 3	7142(±176)	7309(±213)	7588(±202)	6644(±179)
Experimental CCS [Å^2^]^[i]^	6827(±81)	7030(±113)	7173(±68)	7024(±97)
Deglycosylated CCS [Å^2^]^[j]^	6851(±61)	7087(±56)	7202(±43)	7095(±51)
Net solution charge^[k]^	20+	2−	2+	2+

[a] Approximate mass of glycosylated protein given sequence variability in Fc and Fab regions. [b] Experimentally observed glycosylated mass from MS (± standard deviation). [c] Lowest observed experimental charge for glycosylated proteins. [d] Fc to Fab distances (Uniprot: IgG1 P01857, IgG2 P01859, IgG3 P01860, IgG4 P01861). [e] CCS=PA×1.14 calculated by IMPACT for starting models. [f] Lowest CCS_model_ generated from Fab arm sampling. [g] CCS_model_ range of ensemble from Fab arm sampling. [h] CCS_model_ for triplicate models following 10 ns of gas‐phase simulation (± denotes CCS_model_ range over final 1 ns). [i] Average CCS for lowest charge over T‐waves 550, 600, and 640 ms^−1^ (± standard deviation) for glycosylated proteins. [j] Average CCS for lowest charge over T‐waves 550, 600, and 640 ms^−1^ (± standard deviation) for deglycosylated proteins. [k] Net solution charge of each IgG molecule as determined from the Protparam webserver (Supplementary Figure 2).

The ability of significant compaction is likely provided by IgG hinges imparting the steric freedom necessary for the Fab and Fc domains to contort into a compact structure. While collapse of IgG structures and other flexible molecules in the gas phase have been widely observed, simulating their collapsed structures remains a challenge. Previous studies beginning gas phase simulations directly from crystal structures of IgG molecules, show more than 20 % discrepancy from their CCS_exp_ values.^34^, ^35^ Thus, we hypothesise that IgG molecules may experience precollapse prior to transfer into the gas phase.

With the aim of developing a methodology able to simulate the collapse of IgG molecules, we designed an in silico workflow for pairing experimental data with computational models (Figure 1 c). In the first step of our modelling workflow, we subject each of the four initial IgG models to Fab arm conformational sampling using a rapidly exploring random tree algorithm (see Methods in the Supporting Information). This sampling technique produces randomly varied Fab conformations given the degrees of freedom allowed by the antibody's flexible upper hinge residues. Specifically, the conformations of residues between the most N‐terminal hinge disulphide bond and the Fab domains are explored. Sampling in this way produces a model ensemble of conformations of IgG structures which mimic their flexibility in a solution environment.

Through Fab arm sampling, we generated 10 000 conformations for each IgG and calculated the model variation over each ensemble (Figure 2 b, Table 1, Supplementary Figures 11, 12). The CCS variation in each of the IgG ensembles correlated well with previously reported Fab flexibility (IgG3>IgG1>IgG4>IgG2)^6^, ^49^ and upper‐hinge lengths. Closer inspection of the conformational space occupied by each Fab arm revealed that Fabs of IgG1, 2, and 4 are restricted to their own hemispheres (Figure 2 b). The Fabs of IgG3 however, share a high degree of overlapping space indicative of their enhanced flexibility provided by the longer upper‐hinge region (Figure 2 b).

Our sampling strategy identified conformations of IgG1 that are highly similar to those modelled through solution‐based small‐angle X‐ray scattering (SAXS).^50^ A recent study also highlighted the ability of Fab arm sampling and clustering analysis to deliver solution‐relevant antibody conformations.^51^Within our IgG2 ensemble, movement of Fab arms are more confined due to the presence of two extra disulphide bonds located in the upper hinge compared to the other short IgG molecules (IgG1 and IgG4; Table 1). While Fabs of IgG1, IgG3, and IgG4 are able to flex away from their Fc, exposing potential receptor binding sites (Supplementary Figure 1), this dynamic behaviour is not shared to such a degree by IgG2. It is interesting to speculate that this modelling observation may offer insight into why experimentally, IgG2 exhibits reduced affinity to some FcγR receptors compared to IgG1, 3, and 4.^14^, ^52^

While Fab arm sampling provides a powerful method of exploring the conformational space, it is important to note that this is a pseudosimulation and does not take the energetic landscape of the molecule into account. To provide structures relevant to the gas‐phase environment, we subjected models from each ensemble to molecular dynamics (MD) in vacuo (Supplementary Figures 13–16, Methods). We performed simulations in triplicates by selecting the lowest CCS and two low CCS models from each Fab arm sampling ensemble. Each model was precharged with the lowest observed experimental charge state (Table 1). The models were then simulated in vacuo for 10 ns (Methods). All CCS_model_ calculated for the final simulation frame for each IgG were within approximately 6 % of the CCS_exp_ values, with the closest match being IgG3 showing 0.1 % CCS difference. For each set of three simulations, the CCS difference between independent simulations were 3.8 % for IgG1, 1.9 % for IgG2, 4.3 % for IgG3, and 4.9 % for IgG4. We additionally carried out simulations of IgG4 for charge states 22–25+ which show agreement with experimental values (Supplementary Figure 17). To the best of our knowledge, this is the first time that the dynamic structures of substantially collapsed IgG molecules have been modelled with this level of agreement with experimental values. Overlaying the CCS_model_ of our collapsed models with the experimental CCS distribution showed that each individual simulation occupies a narrow CCS range, thus indicating that collapsed structures are relatively inflexible in the gas phase (Figure 2 c). We hypothesise that IgG flexibility in solution leads to a diverse population of rigid collapsed structures in the gas phase, resulting in the observed experimental CCS distribution. The results of each step of our modelling workflow has been summarised in Figure 3.

**Figure 3:**
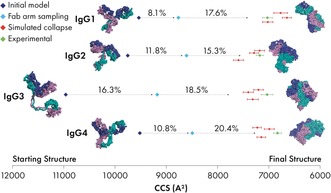
Summary of experimental and model CCS for IgG1–4. CCS was calculated for each stage of the modelling workflow. The reduction in CCS between modelling stages is shown by percentages. Error bars for the experimental data points (green) represent the standard deviation of measurement. Error bars for simulated collapse models (red) show CCS range over the last 1 ns of simulation.

Our modelling workflow has generated collapsed models of IgG1–4 that closely match experimental CCS values. The steps undertaken aim to simulate the collapse of these molecules in the gas phase whilst remaining consistent with the current consensus of the CRM of folded proteins undergoing ESI. The timeline emerging from our workflow envisions that IgG molecules, being flexible in their solution environments, are coerced into semicollapsed conformations by their shrinking droplets. This theory is supported by an MD study which saw gradual desolvation of ubiquitin, cytochrome c, and holo‐myoglobin trajectories over periods longer than 100 ns.^40^ While IgG semicollapsed conformations are accessible through Fab arm sampling, this procedure provides two additional benefits. Firstly, computational timescales can be cut significantly as the IgG has already collapsed to mimic a state in which nearly all of the solvent molecules have already evaporated from the protein. As a result, our gas‐phase MD simulations converge after a much shorter period of time. Secondly, charges cannot be misassigned to surfaces of the protein which later form the collapsed interfaces (which would prevent collapse). Therefore, we speculate that CRM charge transfer occurs concurrently or after partial collapse resulting in charge migration from solvent to exposed protein surfaces. This hypothetical model has been summarised in Figure 4.

**Figure 4:**
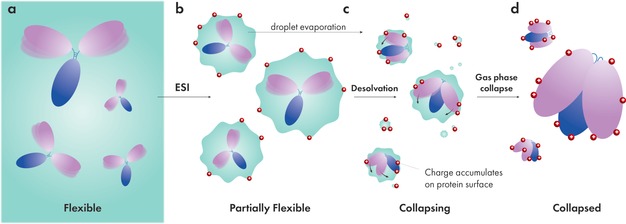
Proposed collapse pathway of IgG during ESI. a) IgG molecules exhibit full flexibility in solution. b) Nanospray ESI produces charged droplets in which IgG molecules retain partial flexibility depending on droplet size. c) Gradual evaporation of droplets coerces flexible IgG molecules into more compact topologies. Solvent charges migrate to protein surfaces as they become exposed through desolvation (CRM). d) Dry protein ions are inflexible in vacuum and represent a distribution of compact conformations.

Probing the conformational dynamics of antibodies by IM‐MS has led to several interesting conclusions because of their intrinsic flexibility. Firstly, the flexibility of IgG molecules can be represented through Fab arm sampling and allow inferences to be made about their solution dynamics. Building on these solution‐relevant conformations, we theorized that IgG molecules undergo partial collapse in solution, which eventually leads to their collapsed topologies in the mass spectrometer. The ability to model these collapsed structures accurately has provided insight into the experimental CCS distribution of these flexible molecules.

In conclusion, this study highlights the need for a predictive model when interpreting gas‐phase data as nonglobular proteins are unlikely to retain their native structures. A combination of high throughput IM‐MS and molecular modelling may therefore provide complementary structural and dynamical information to other biologically relevant techniques such as hydrogen deuterium exchange mass spectrometry. Such approaches may facilitate invaluable data interpretation which might not be possible without structural representations. Ultimately, we anticipate that this workflow will be applicable to other flexible proteins currently eluding solution or gas‐phase structural and dynamical characterization.

## Conflict of interest

The authors declare no conflict of interest.

## Supporting information

As a service to our authors and readers, this journal provides supporting information supplied by the authors. Such materials are peer reviewed and may be re‐organized for online delivery, but are not copy‐edited or typeset. Technical support issues arising from supporting information (other than missing files) should be addressed to the authors.

Supplementary
