# Supplementary material for: A Mass‐Spectrometry‐Based Modelling Workflow for Accurate Prediction of IgG Antibody Conformations in the Gas Phase
Source: Angew Chem Int Ed Engl. 2018 Nov 27;57(52):17194–9. doi: 10.1002/anie.201812018 (PMC6392142; doi:10.1002/anie.201812018)
Supplement: Supplementary file 1 — Supplementary [file ANIE-57-17194-s001.pdf]

Angewandte Chemie
Eine Zeitschrift der Gesellschaft Deutscher Chemiker

Supporting Information

A Mass-Spectrometry-Based Modelling Workflow for Accurate Prediction of IgG Antibody Conformations in the Gas Phase

*Kjetil Hansen[+], Andy M. Lau[+], Kevin Giles, James M. McDonnell, Weston B. Struwe, Brian J. Sutton, and Argyris Politis**

anie_201812018_sm_miscellaneous_information.pdf

Materials and Methods

Sample Preparation

IgG1, IgG2, IgG3 and IgG4 (kappa from human myeloma plasma) were purchased from Sigma-Aldrich at a concentration of 1 mg/mL. Lyophilised intact mAb mass check standard was purchased from Waters. Herceptin was purchased from the Churchill Hospital Pharmacy, University of Oxford. The proteins were buffer exchanged into 150 mM ammonium acetate pH 7 using Micro Bio-Spin columns (Bio-Rad) prior to running on the mass spectrometer. Transthyretin (TTR; 56 kDa), alcohol dehydrogenase (ADH; 148 kDa) and glutamate dehydrogenase (GDH; 336 kDa) were used as CCS calibrants and were buffer exchanged using the same procedure as above. The glycans were removed for the deglycosylation experiments with PNGase F (New England BioLabs) for 4 hours at 37 °C.

Ion Mobility

A commercial qTOF-TWIMS instrument (Synapt G2-Si, Waters) was used with a nano-ESI source. The instrument was run with positive polarity in sensitivity mode and calibrated with caesium iodide. Capillaries were pulled in-house with a Flaming/Brown P-97 micropipette puller (Sutter Instruments) and coated with Au:Pd (80:20) using a sputter coater (Quorum Q150RS). The following mass spectrometer settings were used: capillary voltage 1.3-1.8 kV, sample cone 50 V, source temperature 45 °C, trap pressure $3.6e^{-2}$ mbar, drift tube pressure 2.6 mbar, IMS wave height 40 V, m/z range 500 – 12000, cone gas 0 L/hr. Nitrogen was used as the ion mobility gas and drift times were collected at IMS wave velocities of 550, 600 and 640 ms^{-1}.

The resulting data was processed using MassLynx V4.1 (Waters Corp. Manchester, UK) and PULSAR[1] which contains literature values for the CCS calibrants[2]. CCS_{exp} of TTR, ADH and GDH were used to generate calibration curves (for each T-wave velocity) to which the IgG1-4 data points were fitted (R^2 = 0.985-0.989). Final CCS_{exp} for each IgG1-4 were taken

from the lowest charge state species. All CCS_{exp} values were converted to CCS_{He} in PULSAR.

High-Resolution Native Mass Spectrometry

A Thermo Q-Exactive mass spectrometer (Thermo Fisher Scientific, Germany) modified for detection of high molecular weight ions was used for IgG1 glycosylation analysis. Data was obtained in positive ion mode with an acquisition window of m/z 1000 to 15000. Ions were desolvated in the HCD cell with 100V. Additional settings were as follows: capillary voltage = 0.8-1.0 kV; source temperature = 60°C; max injection time = 100 ms; S-lens RF = 150; resolution = 17500. Spectra were obtained with 10 microscans, averaged over 50 scans. Data was processed using XCalibur 2.1 software (Thermo Fisher Scientific, Germany) and glycoforms were assigned manually.

Generating initial models of IgG1, IgG2 and IgG4

All homology modelling was performed using MODELLER[3]. IgG1 (Uniprot accession: P01834 and P01857) was modelled using PDBs 1HZH (human) and 1IGY (mouse) as template (Supplementary Figure 2). The Fab of 1HZH (chains B and D) missing covalent connection to the rest of the molecule, was extracted and aligned to the Fab of 1IGY in order to recover an extended solution-like conformation of IgG1. The structure of IgG2 (AN: P01834 and P01859) was modelled using PDB 1IGT (mouse; whole molecule), 4L4J (human; Fc) and 2QSC (human; Fabs). Two additional disulphides were inserted into the hinge to generate a representative model of human IgG2. 200 models of each IgG1 and IgG2 were generated and evaluated based on their discrete optimised protein energy (DOPE) score[4]. Missing residues of the human IgG4 crystal structure were re-generated automatically in MODELLER using PDB 5DK3 (human) as template. Glycans structures were not modelled into any of the IgG molecules for two reasons. Firstly, each IgG exhibits numerous glycoforms which dramatically increases the number of starting models of our study, both for the Fab arm sampling and gas phase simulation sections. Secondly,

deglycosylation of IgG molecules results in no significant difference in experimental CCS. These observations have led us to believe that the added complexity of including glycan structures does not offer significant benefits to our modelling workflow.

Homology modelling of IgG3

We acquired fragments of the structure from PDBs 4HAF (human; Fc) and, 4HDI and 1CLZ (mouse; Fab) and manually built the hinge structure using 11 CYS-CYS pairs interspersed with six tri-proline helices (Supplementary Figure 2). All other hinge residues were automatically added with MODELLER. Glycans were not modelled for IgG3, as described in the homology modelling procedure of IgG1, 2 and 4. The IgG3 model was then subjected to 100 ns of explicit solvent molecular dynamics simulation in GROMACS 5.1.3[5] with the CHARMM27 (modified CHARMM22 for proteins) forcefield[6]. The atomistic homology model of IgG3 was added to a triclinic simulation box (178 x 169 x 252 Å) with an edge buffer of 10 Å to account for flexibility and prevent interactions with periodic images. Disulphide bonds were manually checked to ensure correct bonding. 243,611 TIP3 waters and 2 chloride counterions were added to neutralise the system charge. We then performed energy minimisation using a steepest-descent algorithm, followed by equilibration in isochoric-isothermal (300 K, τ = 0.1 ps) and isobaric-isothermal (1.0 bar, τ = 2.0 ps) ensembles for 1ns each. Equilibration employed the "V-rescale" modified Berendsen thermostat and Parrinello-Rahman barostats. The LINCS algorithm was employed to restrain bonds. Finally, production simulation of the system was continued for 100 ns at constant temperature and pressure. For non-covalent interactions, we utilised particle mesh Ewald (PME) with a grid spacing of 0.16 nm for long-range electrostatic interactions, and the Verlet cut-off scheme for Van der Waal calculations. The RMSD evolution of the simulation was monitored and reviewed after 100 ns of simulation to ensure appropriate convergence of the IgG3 structure. To extract a single representative model of IgG3, we clustered models from the final 50 ns of the simulation and identified centroid model of the major conformation.

Fab arm conformational sampling

For conformational sampling of each of the IgG1-4 Fab arms, we first identified the selection of residues which constituted their upper hinges. For each IgG heavy chain, these were, IgG1: D446-T450, IgG2: E437-K439, IgG3: E219-T230, IgG4: E437-P443. The conformational space of each upper hinge and its Fab were then sampled using a rapidly exploring random tree (RRT) algorithm available from the Integrative Modelling Platform (IMP)[7]. This procedure sets the disulphide top-most disulphide of each hinge as the tree root and randomly tests availability for each node (connected atom) to rotate to a new position which is also permissible by the residue's torsional space. Conformations which do not result in steric clashing or overlap are exported as a structure within the ensemble. Both Fab arms are sampled simultaneously with 10,000 models being generated in total for each IgG1-4.

Gas phase molecular dynamics simulations of IgG1-4

Each of the lowest CCS conformations of IgG1-4, including two models selected from the pool of lowest 50 CCS models, were subjected to gas phase molecular dynamics simulations using GROMACS 5.1.3[5]. Since there is no method of determining the experimental charge sites, we pre-charged our IgG models using a localised charge model. This model reflects the lowest observed experimental charge state (21+ for IgG1, IgG2 and IgG4, 22+ for IgG3). Charges were applied to a randomly selected distribution of basic (lysine, histidine and arginine) residues which were found within 5 Å residue depth of the protein surface, using a combination of the DEPTH server[8] and in-house scripts. Pre-charging was repeated where charges were placed too close to each other or prevented the structure from collapsing. We also did not consider acidic residues or neutral salt bridges due to there being no method of accounting for these interactions experimentally. All acidic residues (aspartate and glutamate) remained neutral for our gas phase simulations. Simulations were performed using the OPLS forcefield due to the availability of protonated arginine topologies. All disulphide bonds were manually checked to ensure correct bonding.

Energy minimisation was performed for 50,000 iterations using a steepest descent minimiser, followed by position restraints for all bonds for 500 ps. Simulations were carried out at a temperature of 300 K and regulated using the Berendsen thermostat (τ = 0.1 ps). Pressure coupling and periodic boundary conditions were switched off due to the *in vacuo* nature of the simulations. A cut-off scheme of infinite distance was used for coulombic and van der Waals interactions. Each model was equilibrated briefly at the correct temperature for 1 ns and then for a further 10 ns to produce the collapsed topologies. RMSD, radius of gyration and CCS was monitored throughout all simulations.

Gas phase simulations of IgG4 for charge states 22-25+

All simulations were carried out as detailed above. Each simulation begins from an identical pre-collapsed model of IgG4 (produced by Fab arm sampling). 22, 23, 24 and 25 charge sites were selected randomly using in house scripts. The charge site distributions are different between each simulation. Each simulation was performed for a total of 10 ns, and the average CCS and CCS variation over the last 1 ns of simulation time was calculated.

CCS Calculation of Computational Models

All CCS for IgG structures were calculated as CCS_{He} using IMPACT software[9]. CCS were calculated through scaling the projection approximation (PA) from IMPACT, by a factor of 1.14 to account for PA underestimation. PA measurements from IMPACT have been calibrated to values calculated from the trajectory method, with a root mean square relative error of less than 1%[9]. This linear scaling factor of PA has shown success with approximating the experimental CCS of large protein complexes[10]. While other direct CCS approximation methods such as exact hard sphere scattering (EHSS)[11], trajectory method (TJM)[12] and projection superposition approximation (PSA)[13] are available, the magnitude of models generated in our study (minimum of 50,000), required a high throughput calculation method such as IMPACT[9].

Supplementary Table 1. Experimental values for IgG1 Samples

Subclass[a]	IgG1 from Sigma	Herceptin	Waters mAb standard
Theoretical mass (kDa)[a]	150	150	150
Experimental mass (Da)[b]	149,328 (±89)	148,620 (±64)	149,719 (±54)
Experimental charge[c]	21+	21+	20+
Glycosylated CCS (Å^2)	6827 (±81)	6875 (±50)	6883 (±57)
Deglycosylated CCS (Å^2)	6851 (±61)	6786 (±54)	6762 (±63)

[a] Approximate mass of glycosylated protein given glycoform variability, and sequence variability in Fc and Fab regions for Sigma IgG1. **[b]** Experimentally observed glycosylated mass via MS (± standard deviation). **[c]** Lowest observed experimental charge. **[d]** Average CCS for lowest charge over T-waves 550, 600 and 640 ms^{-1} (± standard deviation) for glycosylated proteins. **[e]** Average CCS for lowest charge over T-waves 550, 600, and 640 ms^{-1} (± standard deviation) for deglycosylated proteins.

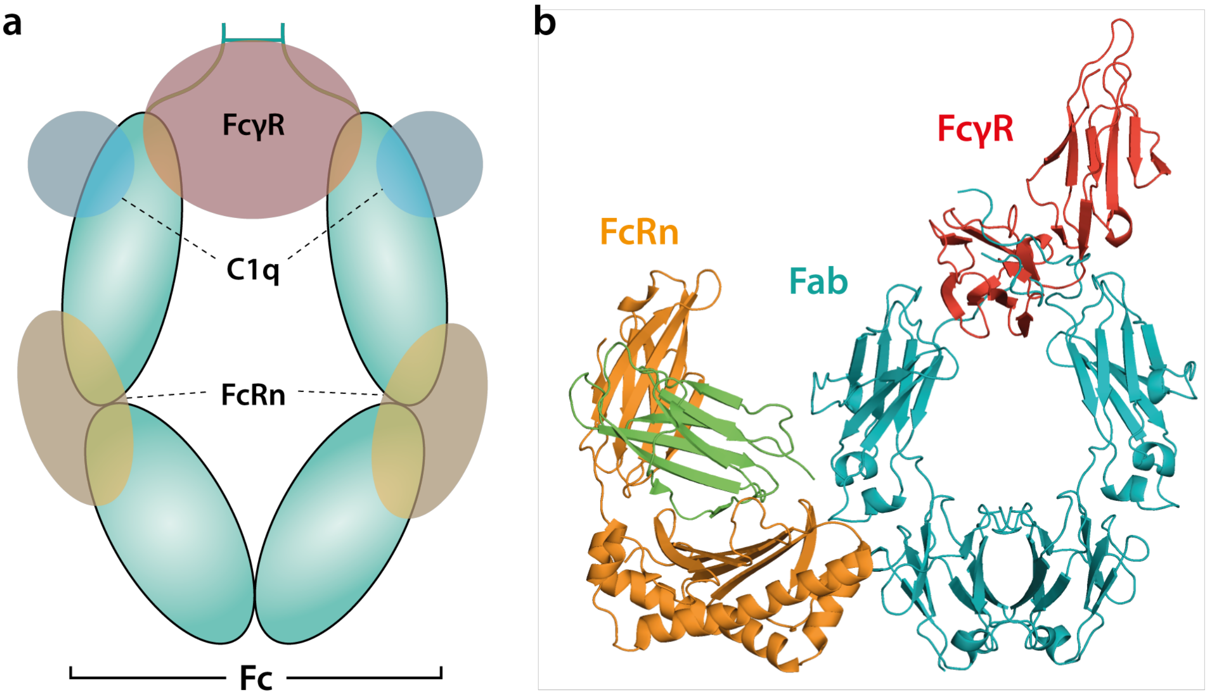

Supplementary Figure 1. Schematic of IgG Fc binding sites. (a) FcγR, C1q and FcRn binding sites are shown for the IgG Fc. (b) Alignment of FcγR crystal structure (orange/green; PDB 1T83) and FcRn (red; PDB 1FRT) to Fc of IgG2 (teal; PDB 1IGT). The hinge is located at the top of the Fc for both (a) and (b). C1q-Fc complex not shown due to the lack of crystallographic representation.

IgG1 PDB: 1HZH | Uniprot: P01857 | Uniprot: P01834 | Acidic: 61 Basic: 71 Net charge: 20+

Heavy

```
QVQLVQSGAEVKKPGASVKVSCQASGYRFSNFVIHWVRQAPGQRFEWMGWINPYNGNKEFSAKFQDRVTFTADTSANTAY
MELRSLRSADTAVYYCARVGPYSWDDSPQDNYYMDVWGKGTTVIVSSASTKGPSVFPLAPSSKSTSGGTAALGCLVKDYF
PEPVTVSWNSGALTSGVHTFPAVLQSSGLYSLSSVVTVPSSSLGTQTYICNVNHKPSNTKVDKKVEPKSCDKTHTCPPCP
APELLGGPSVFLFPPKPKDTLMISRTPEVTCVVVDVSHEDPEVKFNWYVDGVEVHNAKTKPREEQYNSTYRVVSVLTVLH
QDWLNGKEYKCKVSNKALPAPIEKTISKAKGQPREPQVYTLPPSRDELTKNQVSLTCLVKGFYPSDIAVEWESNGQPENN
YKTTPPVLDSDGSFFLYSKLTVDKSRWQQGNVFSCSVMHEALHNHYTQKSLSLSPGK
```

Light

```
EIVLTQSPGTLSLSPGERATFSCRSSHSIRSRRVAWYQHKPGQAPRLVIHGVSNRASGISDRFSGSGSGTDFTLTITRVE
PEDFALYYCQVYGASSYTFGQGTKLERKRTVAAPSVFIFPPSDEQLKSGTASVVCLLNNFYPREAKVQWKVDNALQSGNS
QESVTEQDSKDSTYSLSSTLTLSKADYEKHKVYACEVTHQGLSSPVTKSFNRGEC
```

IgG2 PDB: 1IGT | Uniprot: P01859 | Uniprot: P01834 | Acidic: 60 Basic: 59 Net charge: 2-

Heavy

```
QVQLVQSGGGLVQPGGSLRLSCAAFGFNFSSYVMHWVRQAPGQGLEYLSAISSDGETTYHANSVKGRFTSSRDNSKNTLF
LQMGSLRTEDVAVYYCARDRYYETSGSNAFDVWGQGTMVVVSSASTKGPSVFPLAPCSRSTSESTAALGCLVKDYFPEPV
TVSWNSGALTSGVHTFPAVLQSSGLYSLSSVVTVPSSNFGTQTYTCNVDHKPSNTKVDKTVERKCCVECPPCPAPPVAGP
SVFLFPPKPKDTLMISRTPEVTCVVVDVSHEDPEVQFNWYVDGVEVHNAKTKPREEQFNSTFRVVSVLTVVHQDWLNGKE
YKCKVSNKGLPAPIEKTISKTKGQPREPQVYTLPPSREEMTKNQVSLTCLVKGFYPSDISVEWESNGQPENNYKTTPPML
DSDGSFFLYSKLTVDKSRWQQGNVFSCSVMHEALHNHYTQKSLSLSPGK
```

Light

```
NSVLTQSPSSLSASVGDRVTITCQASQDISNYLNWYQHKPGKAPKLLIYTASNLETGVPSRFSGGGSGTHFSFTITSLQP
EDAATYFCQQYDNLGDLSFGGGTKVEIKRTVAAPSVFIFPPSDEQLKSGTASVVCLLNNFYPREAKVQWKVDNALQSGNS
QESVTEQDSKDSTYSLSSTLTLSKADYEKHKVYACEVTHQGLSSPVTKSFNRGEC
```

IgG3 PDB: 1CLZ | Uniprot: P01860 | Uniprot: P01834 | Acidic: 70 Basic: 71 Net charge: 2+

Heavy

```
EVNLVESGGGLVQPGGSLKVSCVTSGFTFSDYYMYWVRQTPEKRLEWVAYISQGGDITDYPDTVKGRFTISRDNAKNSLY
LQMSRLKSEDTAMYYCARGLDDAGAWFAYWGQGTLVTVSSASTKGPSVFPLAPCSRSTSGGTAALGCLVKDYFPEPVTVS
WNSGALTSGVHTFPAVLQSSGLYSLSSVVTVPSSSLGTQTYTCNVNHKPSNTKVDKRVELKTPLGDTTHTCPRCPEPKSC
DTPPPCPRCPEPKSCDTPPPCPRCPEPKSCDTPPPCPRCPAPELLGGPSVFLFPPKPKDTLMISRTPEVTCVVVDVSHED
PEVQFKWYVDGVEVHNAKTKPREEQYNSTFRVVSVLTVLHQDWLNGKEYKCKVSNKALPAPIEKTISKTKGQPREPQVYT
LPPSREEMTKNQVSLTCLVKGFYPSDIAVEWESSGQPENNYNTTPPMLDSDGSFFLYSKLTVDKSRWQQGNIFSCSVMHE
ALHNRFTQKSLSLSPGK
```

Light

```
DVLMTQIPVSLPVSLGDQASISCRSSQIIVHNNGNTYLEWYLQKPGQSPQLLIYKVSNRFSGVPDRFSGSGSGTDFTLKI
SRVEAEDLGVYYCFQGSHVPFTFGSGTKLEIKRTVAAPSVFIFPPSDEQLKSGTASVVCLLNNFYPREAKVQWKVDNALQ
SGNSQESVTEQDSKDSTYSLSSTLTLSKADYEKHKVYACEVTHQGLSSPVTKSFNRGEC
```

IgG4 PDB: 5DK3 | Uniprot: P01861 | Uniprot: P01834 | Acidic: 62 Basic: 63 Net charge: 2+

Heavy

```
QVQLVQSGVEVKKPGASVKVSCKASGYTFTNYYMYWVRQAPGQGLEWMGGINPSNGGTNFNEKFKNRVTLTTDSSTTTAY
MELKSLQFDDTAVYYCARRDYRFDMGFDYWGQGTTVTVSSASTKGPSVFPLAPCSRSTSESTAALGCLVKDYFPEPVTVS
WNSGALTSGVHTFPAVLQSSGLYSLSSVVTVPSSSLGTKTYTCNVDHKPSNTKVDKRVESKYGPPCPPCPAPEFLGGPSV
FLFPPKPKDTLMISRTPEVTCVVVDVSQEDPEVQFNWYVDGVEVHNAKTKPREEQFNSTYRVVSVLTVLHQDWLNGKEYK
CKVSNKGLPSSIEKTISKAKGQPREPQVYTLPPSQEEMTKNQVSLTCLVKGFYPSDIAVEWESNGQPENNYKTTPPVLDS
DGSFFLYSRLTVDKSRWQQGNVFSCSVMHEALHNHYTQKSLSLS
```

Light

```
EIVLTQSPATLSLSPGERATLSCRASKGVSTSGYSYLHWYQQKPGQAPRLLIYLASYLESGVPARFSGSGSGTDFTLTIS
SLEPEDFAVYYCQHSRDLPLTFGGGTKVEIKRTVAAPSVFIFPPSDEQLKSGTASVVCLLNNFYPREAKVQWKVDNALQS
GNSQESVTEQDSKDSTYSLSSTLTLSKADYEKHKVYACEVTHQGLSSPVTKSFNRGEC
```

Supplementary Figure 2. Sequences of IgG1-4 homology models. The source of the variable and cannonical sequences of each IgG heavy and light chain are shown. Variable regions (red) were inherited from crystal structures used for homology modelling for each IgG. Cannonical sequences of the heavy (purple) and light (blue) chains were accessed from Uniprot. The number of acidic and basic residues were calculated via the ProtParam webserver (https://web.expasy.org/protparam/) for each half of the IgG molecule. The net charge displayed is calculated for the whole IgG molecule.

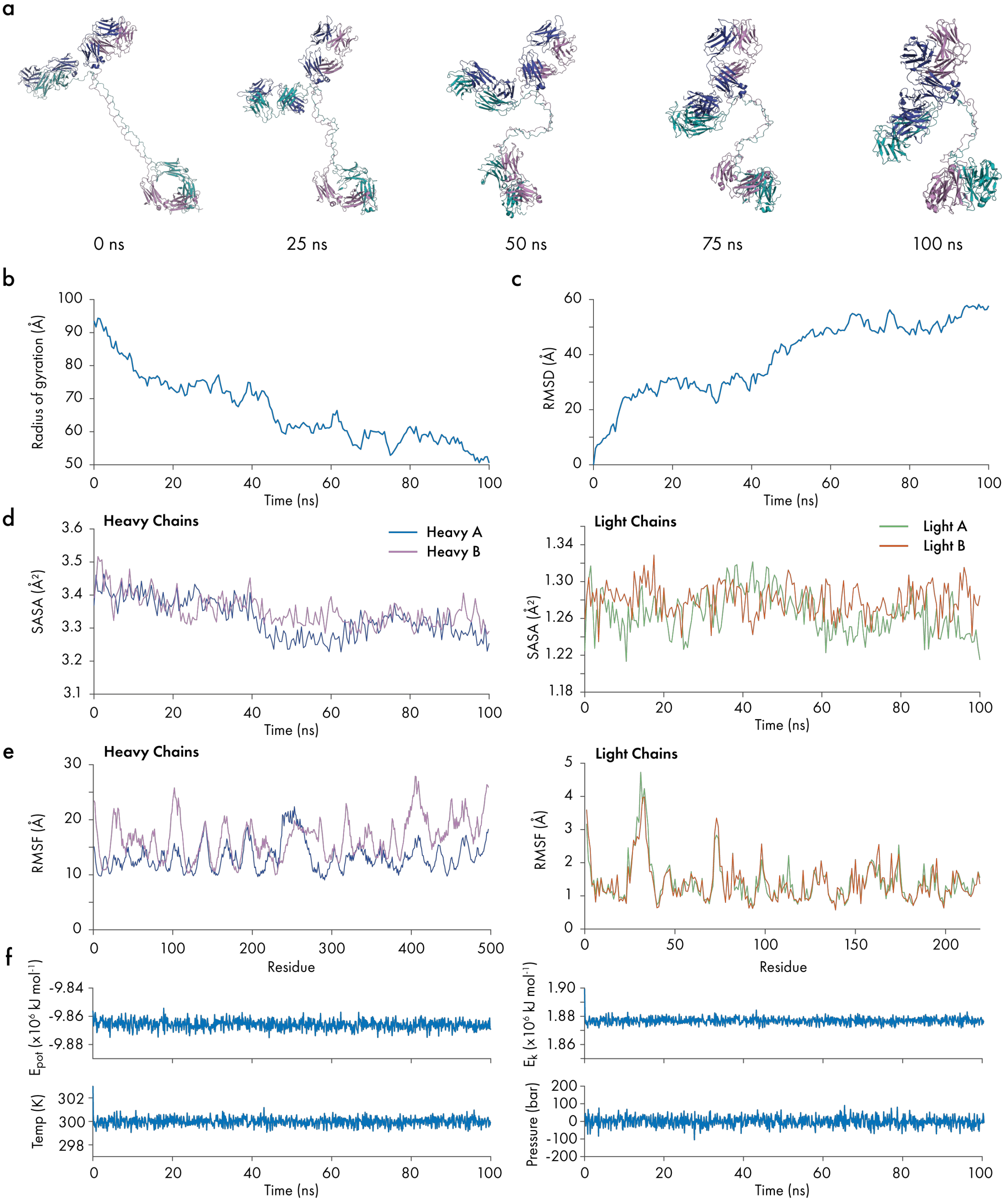

Supplementary Figure 3. Solution simulation of IgG3 homology model. (a) Timeline of IgG3 structure. Heavy chains are shown in teal and violet, light chains are in blue. Simulation parameters (b) radius of gyration, (c) RMSD, (d) solvent accessible surface area (SASA) and (e) root mean squared fluctuation of sidechain atoms over the simulation time. (f) System potential and kinetic energy, temperature and pressure parameters over simulation time. Analysis performed using GROMACS analysis tools[5].

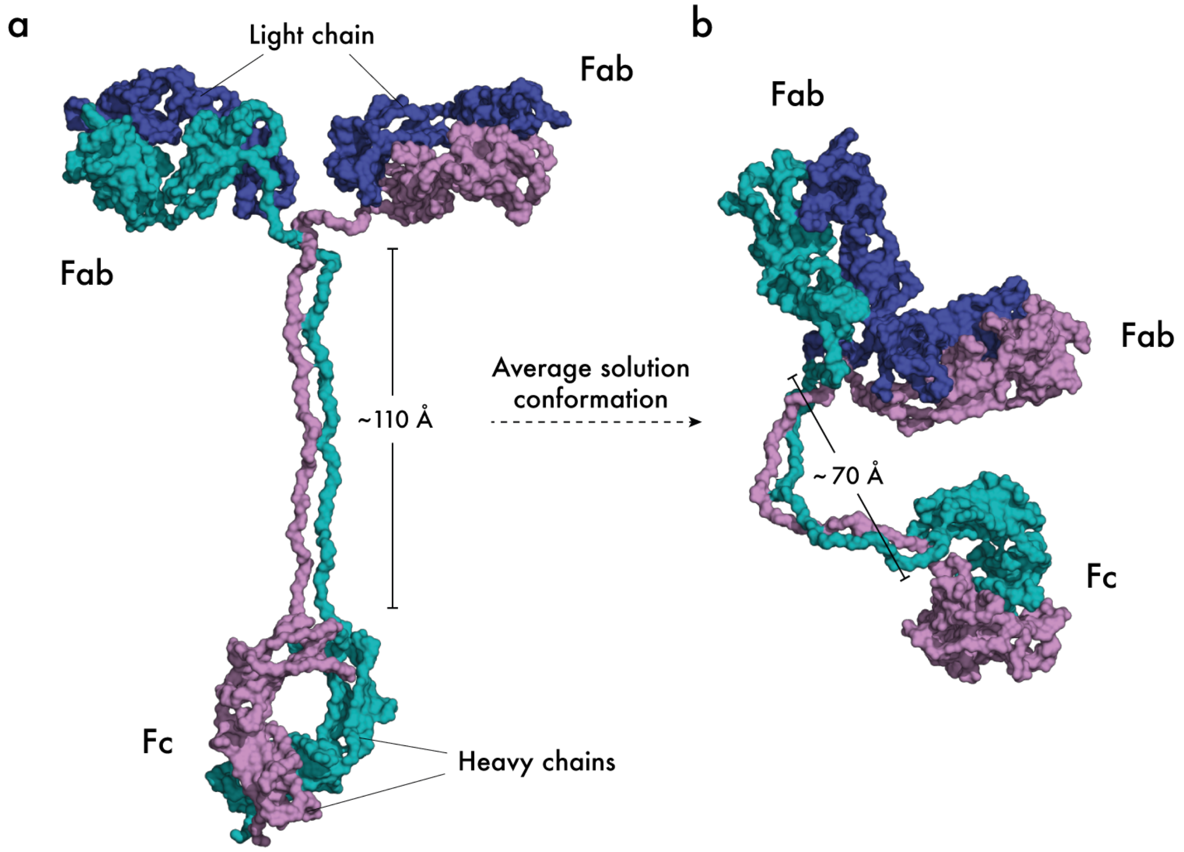

Supplementary Figure 4. Hinge length of human IgG3. (a) Homology model of human IgG3 showing the approximately 110 Å hinge region. (b) Average conformation of human IgG3 following 100 ns of solution simulation showing approximately 70 Å hinge region.

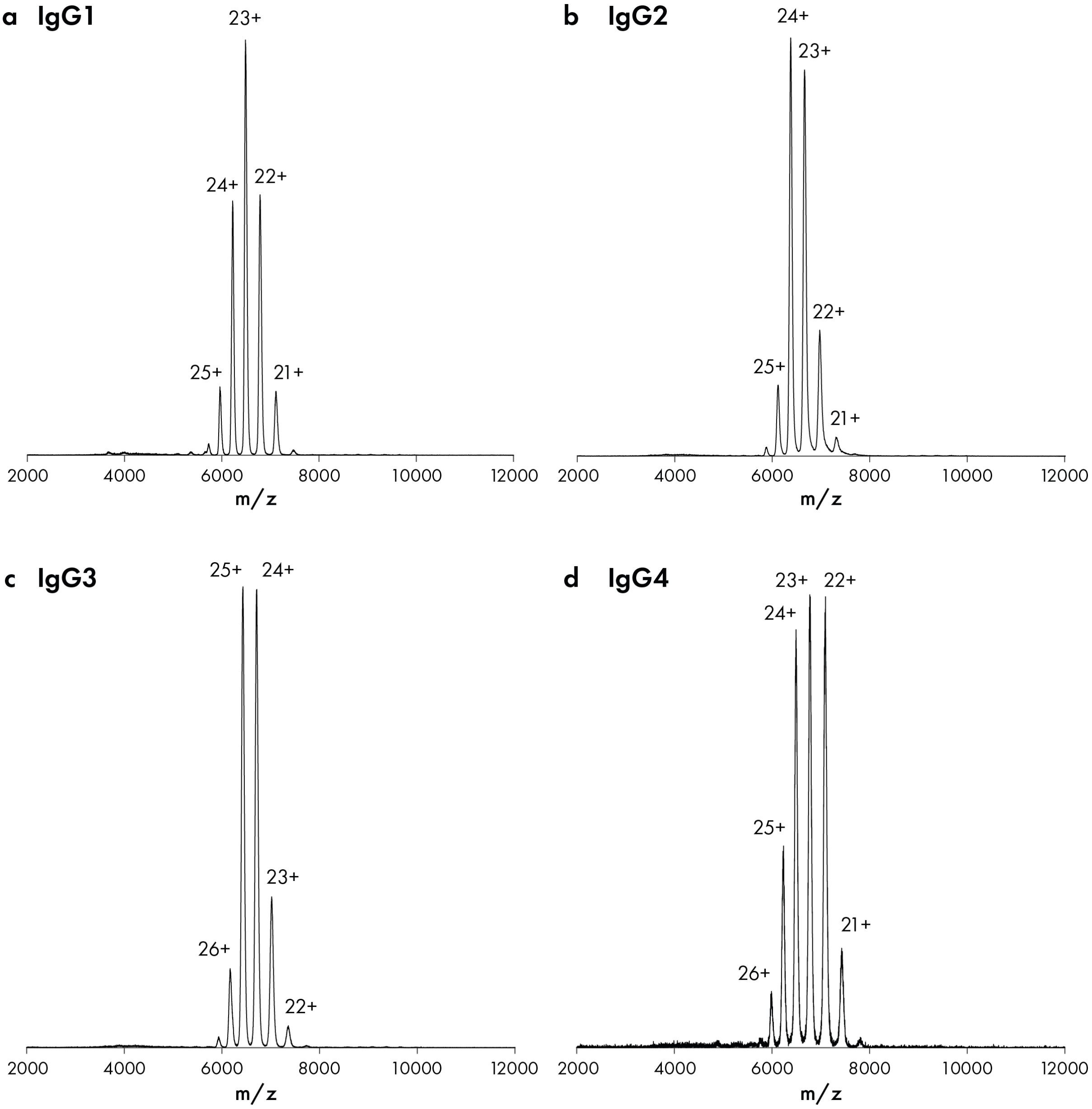

Supplementary Figure 5. MS spectra of glycosylated IgG1-4. Native mass spectra of all four subtypes of glycosylated IgG showing the charge envelope resulting from electrospray ionisation.

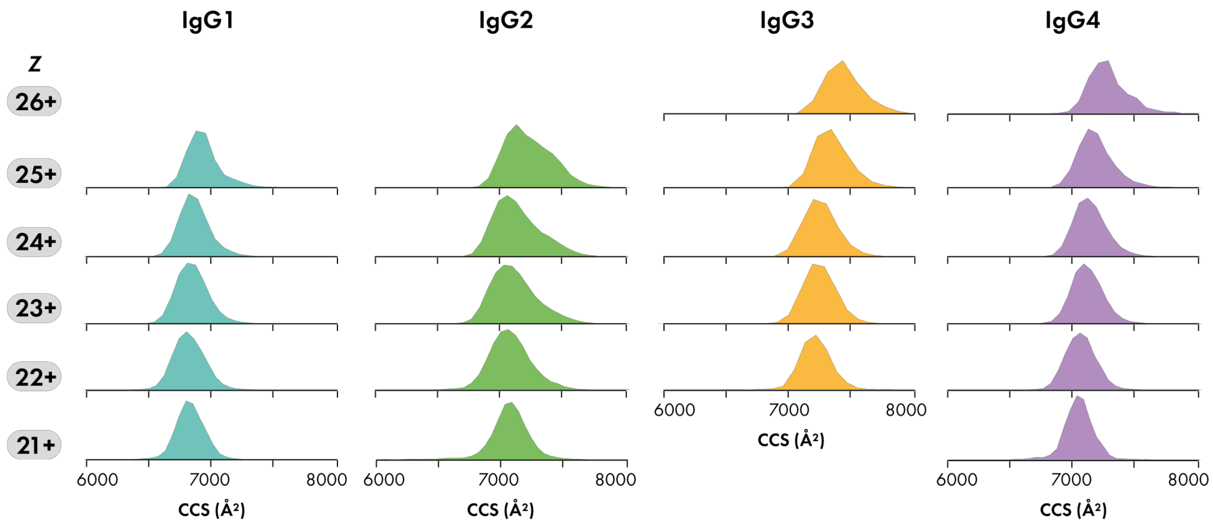

Supplementary Figure 6. CCS distributions of IgG1-4. Collisional cross section distributions for each charge state of IgG1-4 achieved using T-wave ion mobility. Higher charge states exhibit broader distributions.

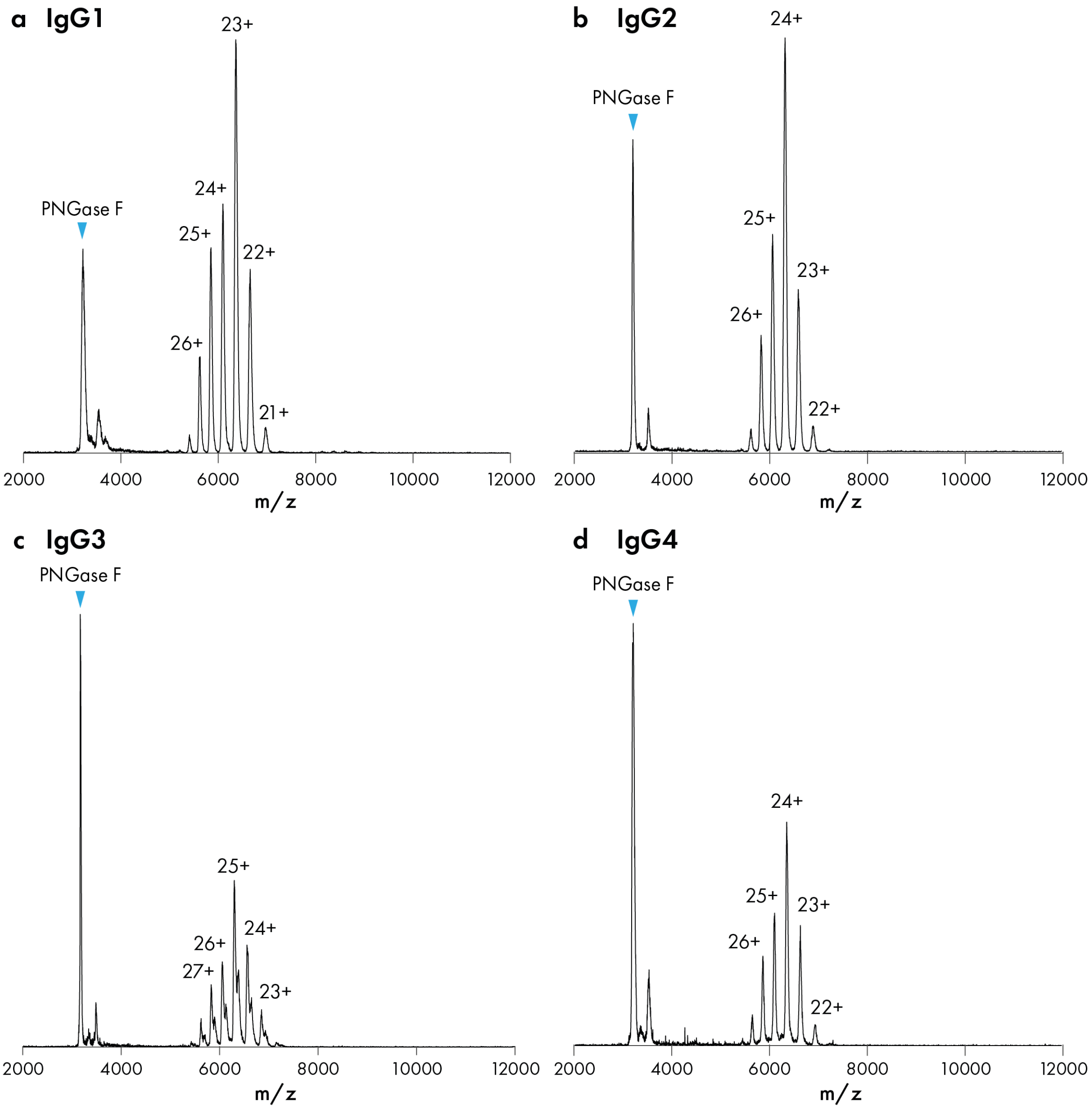

Supplementary Figure 7. MS spectra of deglycosylated IgG1-4. Native mass spectra of all four subtypes of deglycosylated IgG showing charge envelope resulting from electrospray ionisation. The deglycosylation enzyme (PNGase F) has been labelled at approximately m/z 3000.

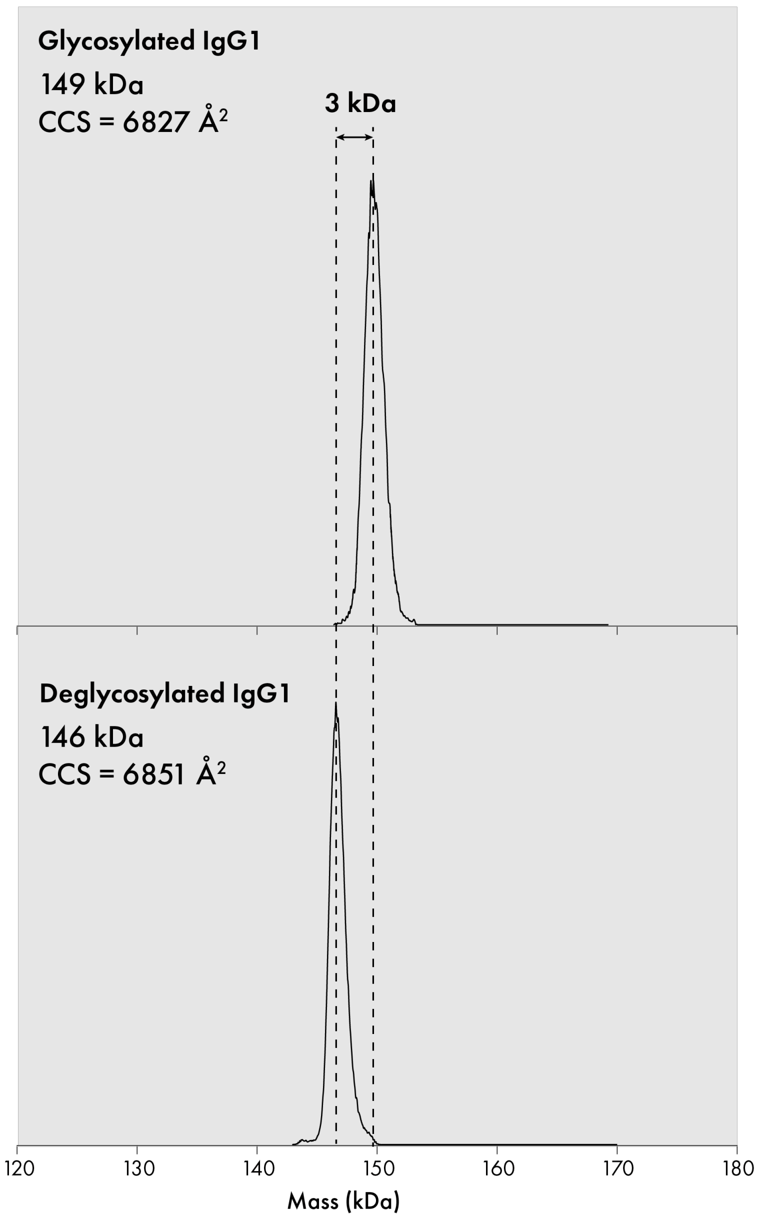

Supplementary Figure 8. Deconvoluted MS spectra of glycosylated and deglycosylated IgG1. Deconvolution performed using Waters MassLynx mass measurement tool and transform function. Mass shift of approximately 3 kDa is observed with no significant changes in CCS.

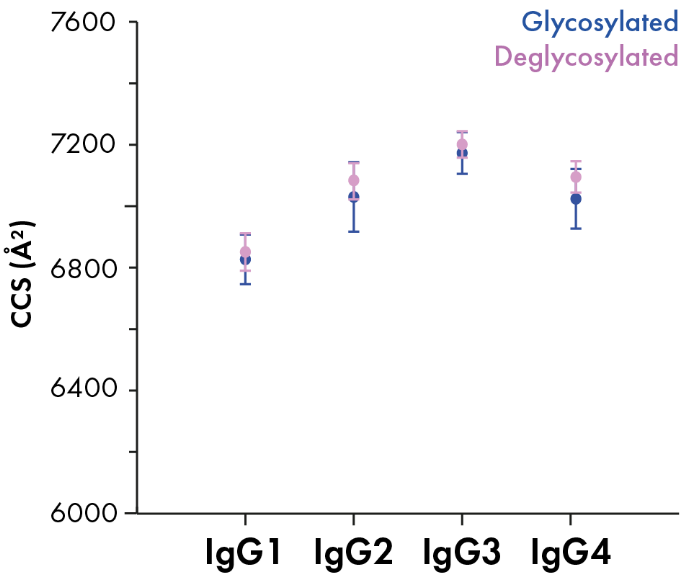

Supplementary Figure 9. CCS_{exp} of IgG1-4. CCS_{exp} for IgG1-4 shown for the lowest experimental charge states (21+ for IgG1, IgG2 and IgG4, 22+ for IgG3). Measurements were recorded and averaged for 550, 600 and 640 ms^{-1} T-wave velocities. Error bars show standard deviations of measurement across T-wave triplicates.

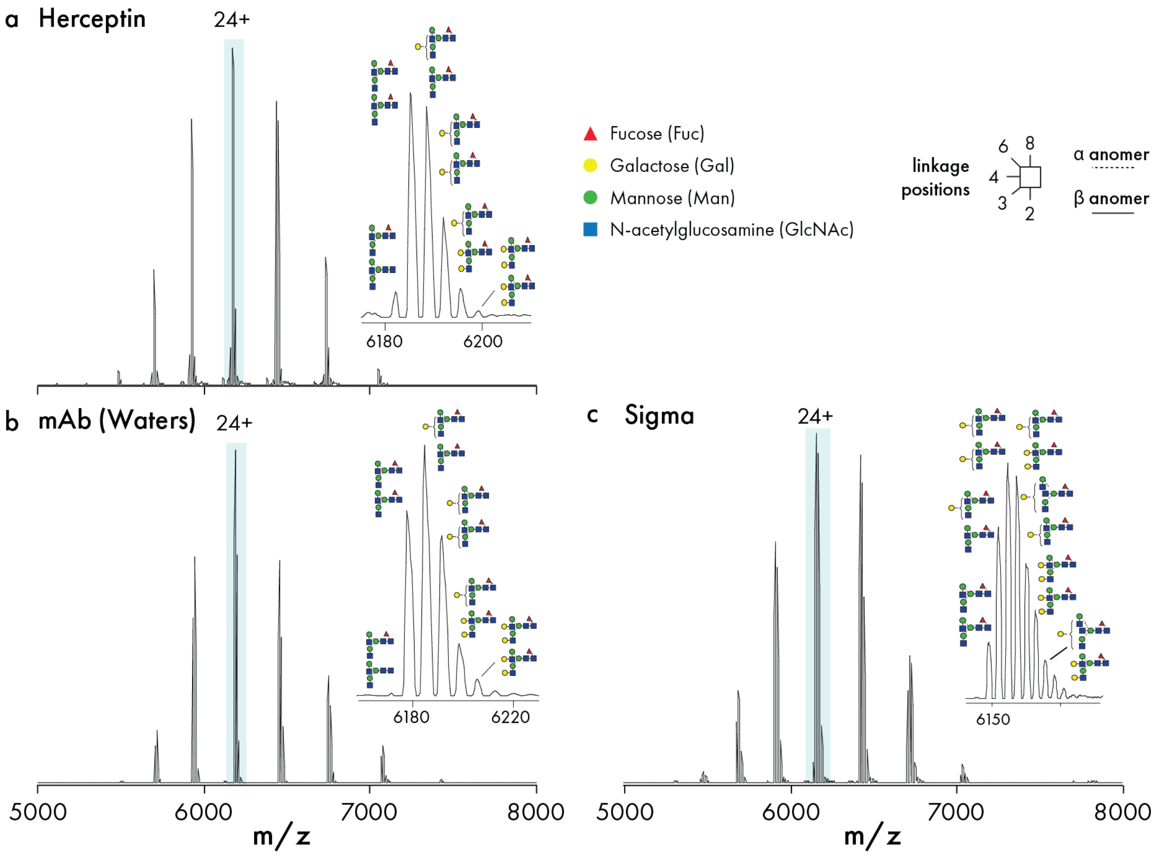

Supplementary Figure 10. High resolution native MS of Herceptin, Waters mAb and Sigma human plasma IgG1 samples, revealing glycoforms heterogeneity. The individual glycoforms are displayed for the most abundant ion (inset). N-glycan structures have been labelled for each identified peak.

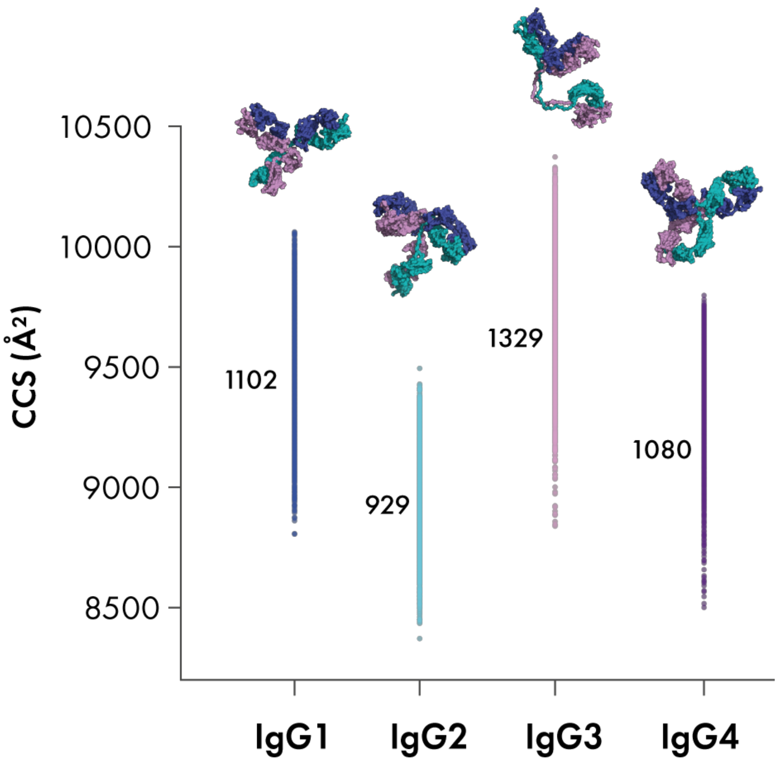

Supplementary Figure 11. CCS of all Fab conformations of IgG1-4 post-sampling. Values indicate the difference between largest and smallest CCS models (ΔCCS). All CCS calculated using IMPACT software[9].

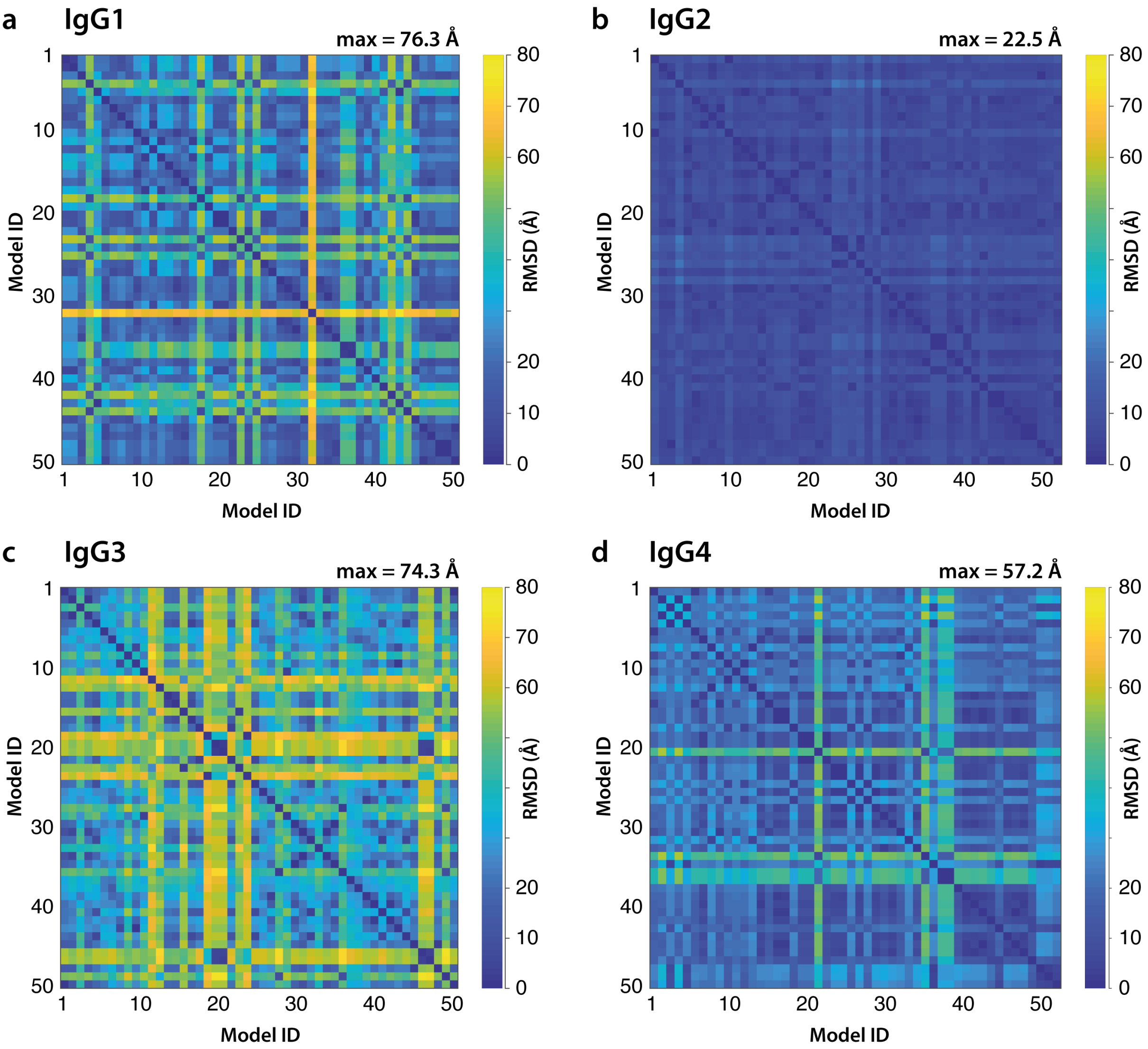

Supplementary Figure 12. RMSD matrices of 50 lowest CCS models of (a) IgG1, (b) IgG2, (c) IgG3 and (d) IgG4. Low RMSD variation between lowest CCS models of IgG2 compared to IgG1, IgG3 and IgG4 suggest a more confined conformational space populated by homogenous models. IgG3 model ensemble exhibits high RMSD variation frequently greater than 70Å, indicating compact conformations can be varied. RMSD calculated between non-fitted Cα atoms only. Max RMSD is shown for each protein.

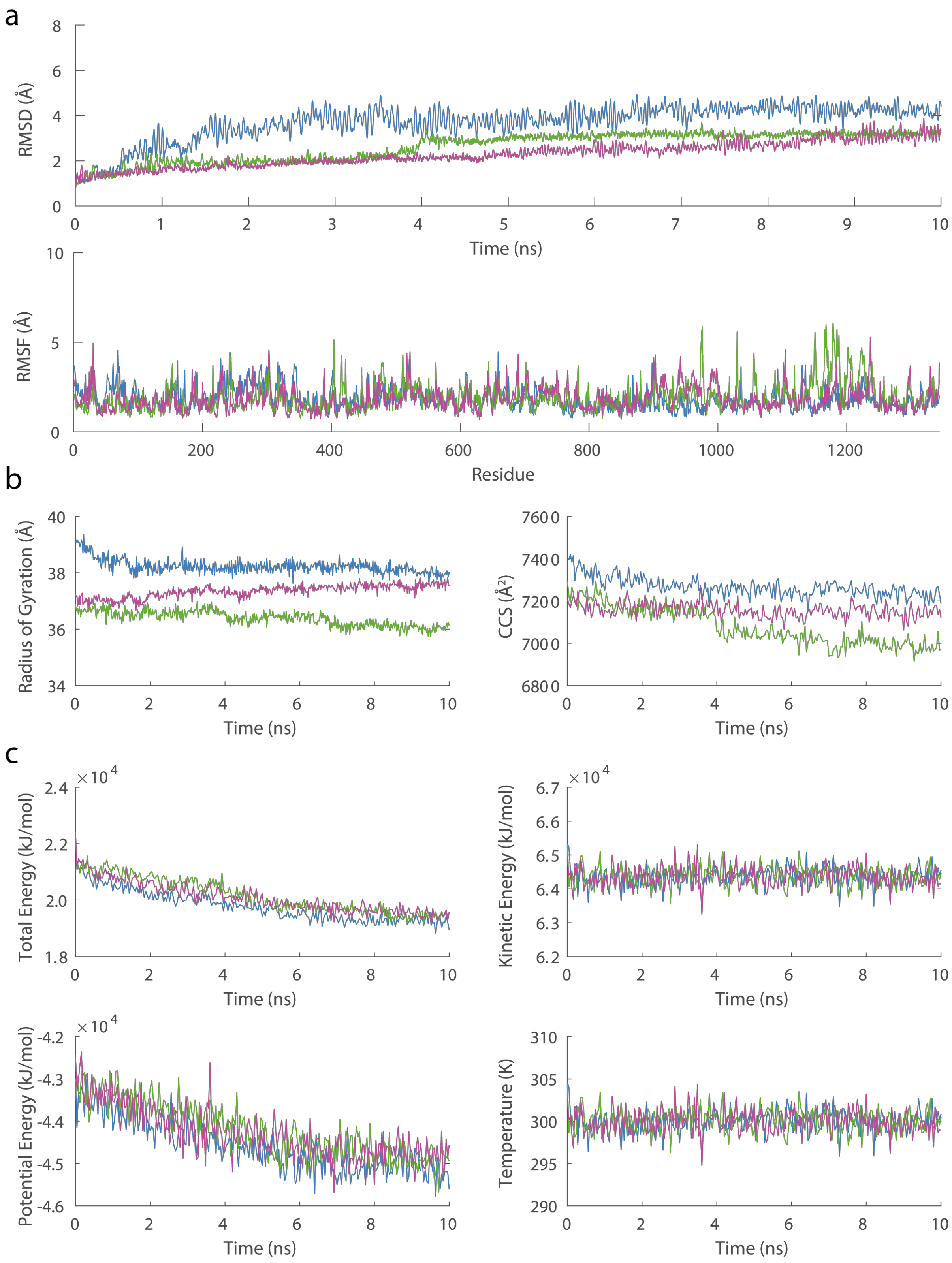

Supplementary Figure 13. Analysis of IgG1 gas phase simulations. (a) Structure similarity measurements RMSD and RMSF, (b) size measurements radius of gyration and CCS, and (c) system parameters over 10ns simulation trajectories for each model.

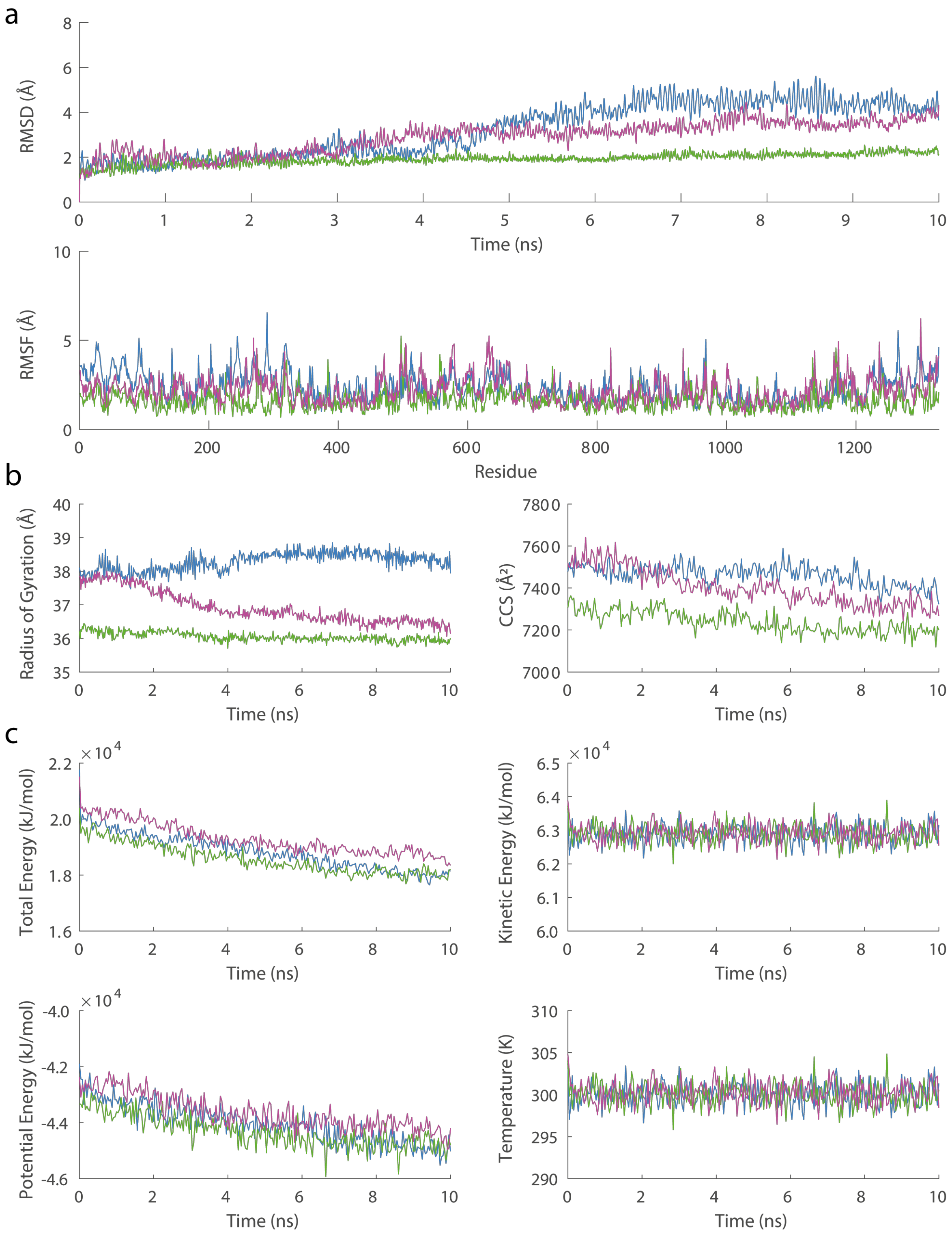

Supplementary Figure 14. Analysis of IgG2 gas phase simulations. (a) Structure similarity measurements RMSD and RMSF, (b) size measurements radius of gyration and CCS, and (c) system parameters over 10ns simulation trajectories for each model.

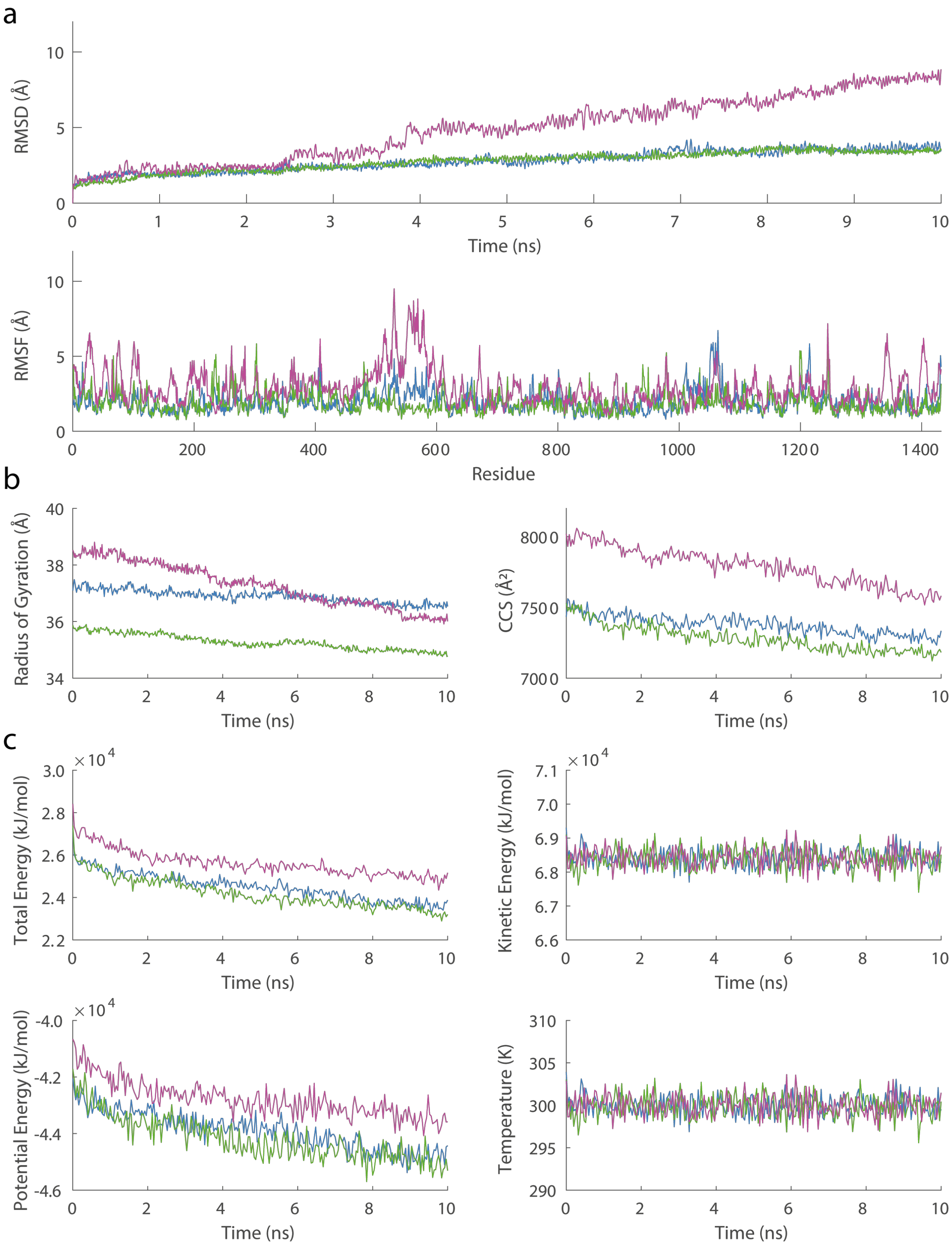

Supplementary Figure 15. Analysis of IgG3 gas phase simulations. (a) Structure similarity measurements RMSD and RMSF, (b) size measurements radius of gyration and CCS, and (c) system parameters over 10ns simulation trajectories for each model.

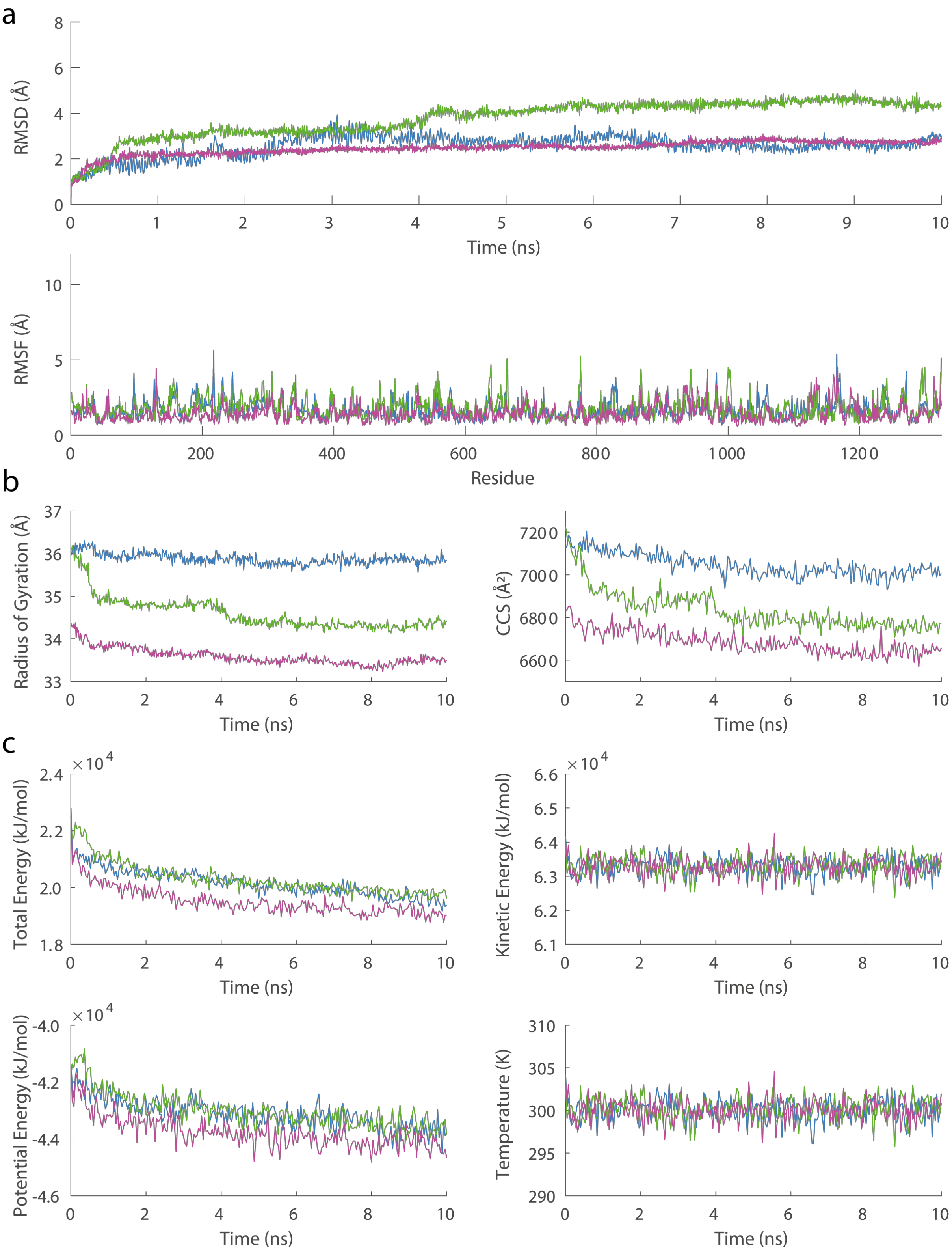

Supplementary Figure 16. Analysis of IgG4 gas phase simulations. (a) Structure similarity measurements RMSD and RMSF, (b) size measurements radius of gyration and CCS, and (c) system parameters over 10ns simulation trajectories for each model.

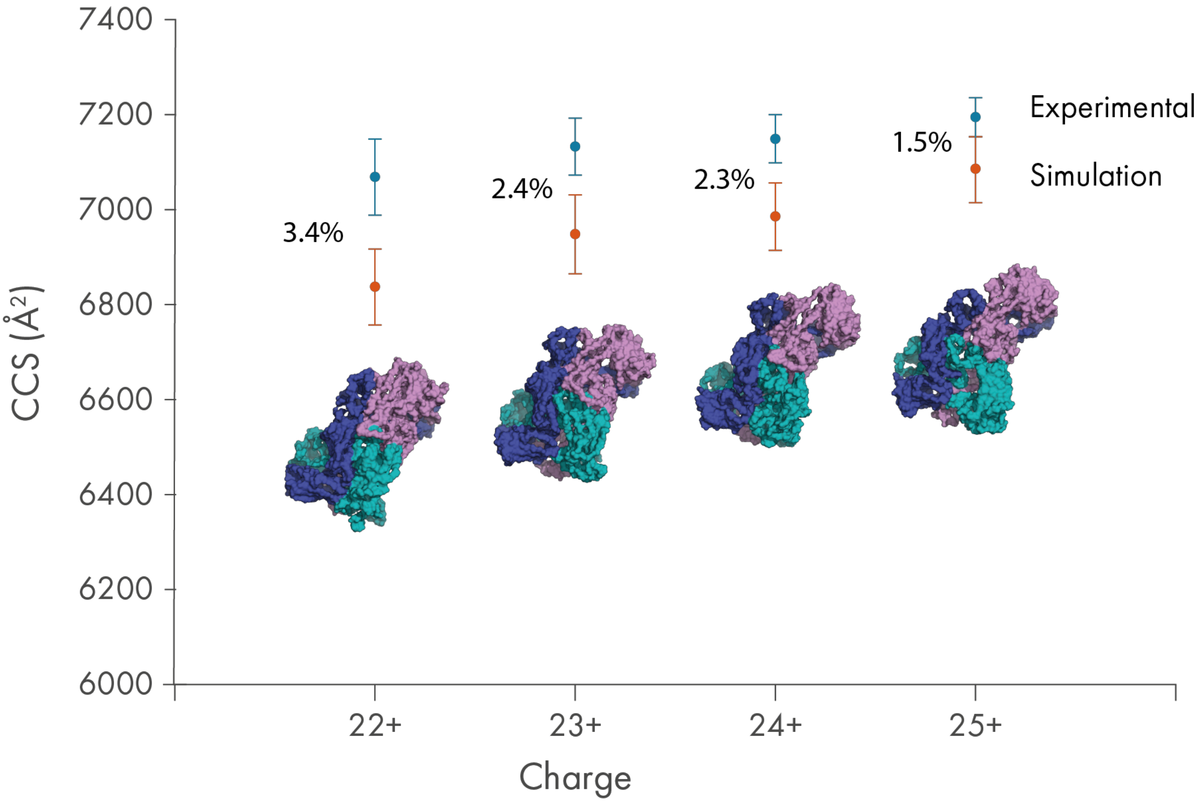

Supplementary Figure 17. Gas phase simulations of IgG4 for charges 22-25+. Beginning from an identical IgG4 model, we performed four simulations, reflecting 22-25+ experimental charge states. Each model was pre-charged using a different random distribution of charge sites and simulated for 10 ns. The average CCS and CCS variation over the last 1 ns of each simulation is shown by the orange data point and error bars. The experimental CCS and standard deviation is shown in blue. The final model of each simulation is shown under each data point. The percentages represent the CCS percentage difference between the experimental and simulated CCS values.

References

[1] T. M. Allison, E. Reading, I. Liko, A. J. Baldwin, A. Laganowsky, C. V. Robinson, *Nat Commun* **2015**, *6*, 8551.

[2] M. F. Bush, Z. Hall, K. Giles, J. Hoyes, C. V. Robinson, B. T. Ruotolo, *Anal Chem* **2010**, *82*, 9557-9565.

[3] A. Fiser, R. K. Do, A. Sali, *Protein Sci* **2000**, *9*, 1753-1773.

[4] M. Y. Shen, A. Sali, *Protein Sci* **2006**, *15*, 2507-2524.

[5] H. J. C. Berendsen, D. Vanderspoel, R. Vandrunen, *Comput Phys Commun* **1995**, *91*, 43-56.

[6] A. D. MacKerell, D. Bashford, M. Bellott, R. L. Dunbrack, J. D. Evanseck, M. J. Field, S. Fischer, J. Gao, H. Guo, S. Ha, D. Joseph-McCarthy, L. Kuchnir, K. Kuczera, F. T. Lau, C. Mattos, S. Michnick, T. Ngo, D. T. Nguyen, B. Prodhom, W. E. Reiher, B. Roux, M. Schlenkrich, J. C. Smith, R. Stote, J. Straub, M. Watanabe, J. Wiorkiewicz-Kuczera, D. Yin, M. Karplus, *J Phys Chem B* **1998**, *102*, 3586-3616.

[7] D. Russel, K. Lasker, B. Webb, J. Velazquez-Muriel, E. Tjioe, D. Schneidman-Duhovny, B. Peterson, A. Sali, *PLoS Biol* **2012**, *10*, e1001244.

[8] K. P. Tan, T. B. Nguyen, S. Patel, R. Varadarajan, M. S. Madhusudhan, *Nucleic Acids Res* **2013**, *41*, W314-321.

[9] E. G. Marklund, M. T. Degiacomi, C. V. Robinson, A. J. Baldwin, J. L. Benesch, *Structure* **2015**, *23*, 791-799.

[10] J. L. Benesch, B. T. Ruotolo, *Curr Opin Struct Biol* **2011**, *21*, 641-649.

[11] A. A. Shvartsburg, M. F. Jarrold, *Chem Phys Lett* **1996**, *261*, 86-91.

[12] M. N. Young, C. Bleiholder, *J Am Soc Mass Spectrom* **2017**, *28*, 619-627.

[13] C. Bleiholder, S. Contreras, M. T. Bowers, *Int J Mass Spectrom* **2013**, *354*, 275-280.
